# Natural Control of Food-Borne Pathogens Using Chitosan

**DOI:** 10.3390/microorganisms13092036

**Published:** 2025-08-31

**Authors:** Gabriella Kiskó

**Affiliations:** Department of Food Microbiology, Hygiene and Safety, Institute of Food Science and Technology, Hungarian University of Agriculture and Life Sciences, Somlói ut 14-16, H-1118 Budapest, Hungary; kisko.gabriella@uni-mate.hu; Tel.: +36-1-305-7360

**Keywords:** chitosan, *Listeria monocytogenes*, *Salmonella*, *Staphylococcus aureus*, *E. coli* O157:H7

## Abstract

The control of micro-organisms in food has a long history. They can be controlled by many different safe methods, including the use of conventional preservatives. In addition, consumers are increasingly distrustful of food processing techniques and the use of preservatives. Therefore, there is a renewed interest and increasing consumer demand for more natural, non-synthesised antimicrobials as potential alternatives to conventional preservatives to control foodborne pathogens and extend the shelf life of foods. Therefore, this review focuses on the application of chitosan as an antimicrobial of animal origin to control major foodborne pathogenic organisms, such as *E. coli* O157:H7, *Listeria monocytogenes*, *Salmonella* sp. and *Staphylococcus aureus*. The antibacterial mechanisms, efficacy, benefits and challenges will be highlighted.

## 1. Introduction

The issue of foodborne illnesses represents a significant concern for consumers, the food industry, and food safety authorities as well. It has been reported that the majority of foodborne illness outbreaks are caused by known pathogens, including but not limited to Norovirus, *Campylobacter* and *Salmonella* species, *Listeria monocytogenes*, and Shiga toxin-producing *Escherichia coli*. In addition, *Staphylococcus aureus*, *Clostridium* species, *Bacillus cereus* and others have been documented as occasional causative agents [1].

The EU One Health zoonoses report [2] indicated that campylobacteriosis and salmonellosis were the two most prevalent zoonoses reported in humans in the EU in 2023, and listeriosis was identified as one of the most severe diseases, with the highest fatality and hospitalisation rates among documented cases. *Salmonella* Enteritidis remained the most frequently reported causative agent in cases of food-borne infections and foodborne outbreaks. The Shiga toxin-producing *Escherichia coli* (STEC), which includes the *E. coli* O157:H7 serotype, was the third most frequently reported zoonotic agent in humans, followed by *Listeria monocytogenes* as the fifth most prevalent agent. The majority of cases of listeriosis for which data is available required hospitalisation (96.5% of confirmed cases). The most common human STEC serogroup was O157 (22.7%). *Salmonella* has been identified as the agent most associated with hospitalisation and fatality. In the case of outbreak scenarios, the most significant case fatality rates were observed in outbreaks attributed to *L. monocytogenes* (8.3%), followed by those caused by the toxin of *Clostridium botulinum* (7.1%) and STEC (0.37%). Among outbreaks caused by bacterial toxins, *Staphylococcus aureus* toxins were the most frequently reported.

Non-typhoidal *Salmonella* species have been identified as one of the leading causes of foodborne diseases in the United States, representing 11% of all reported cases [3,4,5]. Among the pathogens *Listeria monocytogenes* had the highest (94%) domestically acquired foodborne hospitalisation rate with a 15.9% death rate. The hospitalisation and death rates for STEC O157 were 46.2 and 0.5%, respectively, while for non-typhoidal *Salmonella* spp. they were 27.2% and 0.5%, respectively. In contrast, significantly lower hospitalisation and death rates (6.4% and <0.1%, respectively) were reported for food-borne *Staphylococcus aureus* [3].

In both the European Union and the U.S., *Salmonella* spp., verocytotoxin-producing *Escherichia coli* (VTEC), and *Listeria monocytogenes* were among the top five confirmed foodborne pathogens responsible for infections, hospitalisations, and fatalities [4].

The recent focus on discovering natural antimicrobial agents that can prevent the growth of microorganisms has gained considerable interest. The aim of this inquiry is to explore the potential of such agents to enhance food safety and to increase the quality of food products and extend their shelf life. The use of natural antimicrobials as a safer and more sustainable alternative to synthetic ones is being explored [6]. Their utilisation is in line with present consumer demands for healthier and more eco-friendly foods [7].

Natural antimicrobials can be found in plants (e.g., essential oils, extracts), animals (e.g., enzymes, peptides), and microorganisms (e.g., bacteriocins). These antimicrobials can inhibit or kill microorganisms such as bacteria, fungi, and viruses, including food-borne pathogenic bacteria [8,9,10,11,12,13].

Chitosan is a natural, biodegradable, cationic polysaccharide obtained by the partial deacetylation of chitin, which is primarily sourced from shellfish exoskeletons and, more recently, insects [14,15]. It is effective against a broad range of Gram-positive and Gram-negative bacteria, including *Staphylococcus aureus*, *Listeria monocytogenes*, *Escherichia coli*, *Salmonella* spp., *Pseudomonas aeruginosa*, *Proteus mirabilis*, *Sarcina* spp., *Enterococcus faecalis*, *Vibrio cholerae*, *Shigella dysenteriae*, and various fungi and yeasts [16,17,18,19,20,21,22,23,24]. The antibacterial effectiveness of chitosan against Gram-positive and Gram-negative bacteria remains somewhat controversial. A number of studies have reported that chitosan generally exhibits stronger activity against Gram-positive bacteria, including *Listeria monocytogenes*, *Bacillus megaterium*, *Bacillus cereus*, *Staphylococcus aureus*, and lactic acid bacteria such as *Lactiplantibacillus plantarum* and *Levilactobacillus brevis*, when compared to Gram-negative strains such as *Escherichia coli*, *Pseudomonas fluorescens*, *Salmonella* Typhimurium, and *Vibrio parahaemolyticus* [25,26,27,28]. However, other findings suggest that Gram-negative bacteria, due to their higher hydrophilicity, may actually be more susceptible to treatment with chitosan [29]. The validity of this statement is supported by other in vitro studies which demonstrate that Gram-negative bacteria frequently undergo more pronounced morphological alterations upon exposure to chitosan compared to Gram-positive bacteria [30,31,32]. A pivotal factor influencing the antibacterial activity of chitosan is the charge density on the bacterial cell surface, which determines the extent of adsorption. Greater adsorption of chitosan has been shown to result in more significant alterations in cell membrane structure and permeability. These observations suggest that the antibacterial mechanism of chitosan is contingent on the specific characteristics of the target microorganism [33].

Besides antimicrobial action, chitosan exhibits several biological activities including anti-inflammatory, immune-modulating, anti-allergic, anti-hypertensive, antidiabetic, hypolipidemic and hypocholesterolemic, as well as anticoagulant, antioxidative, neuroprotective and wound-healing effects [34,35,36,37,38].

Chitosan is widely used due to its biocompatibility, abundance, and non-toxic nature. It is frequently used in food industry, for example, as an edible antimicrobial coating, to extend shelf life of various foods such as fish, meat, and cheese [39,40,41,42,43,44]. In the field of agriculture, it enhances fruit preservation and plant disease resistance [45]. In the medical and pharmaceutical areas, it is applied in wound dressings, drug delivery, and as an immune enhancer [46,47,48]. In addition, it is also used in water treatment for the removal of dyes, pharmaceuticals, and toxic metals from wastewater [49,50].

This article aimed to review the potential food applications of chitosan, an antimicrobial agent of animal origin, in enhancing the shelf life and safety of food. The focus was on the control of four major foodborne pathogenic bacteria, namely *Listeria monocytogenes*, *Staphylococcus aureus*, *E. coli* O157:H7 and *Salmonella* spp.

## 2. General Properties of Chitosan

The term “chitosan” refers to a diverse group of structurally different chemical entities [51]. It is a natural, biodegradable, and biocompatible polymer derived from chitin, the second most abundant biopolymer on Earth after cellulose [52,53,54,55]. It is composed of D-glucosamine and N-acetyl-D-glucosamine units, formed through partial or complete deacetylation of chitin [56,57,58]. Its physicochemical and biological properties (solubility, antimicrobial activity, film formation) are dependent on several factors such as degree of deacetylation, molecular weight, source, and extraction method [58,59,60,61].

Chitosan is positively charged in acidic environments, which enhances its solubility and antimicrobial activity [58]. It exhibits broad-spectrum antimicrobial and antioxidant properties by several mechanisms including the disruption of microbial membranes, the chelation of metal ions, and the scavenging of free radicals [62,63].

It is applied in many fields including agriculture [64,65,66], biomedicine [67,68,69], and cosmetics [70,71]. Chitosan has a wide range of known food applications, among them proven broad-spectrum action in both laboratory settings and real food systems (e.g., pork, blueberries, fish) [26,72,73,74]. In the food industry, it is used as a thickener, and stabiliser [72,75,76]. It is also applied for clarification [62,77,78,79,80], contaminant removal [81,82] microbial control [43,83,84], and shelf life extension [74,85,86,87]. It acts as a natural alternative to sulphur dioxide in winemaking [88,89]. Chitosan is also commonly applied in edible films [90,91,92,93], coatings [94,95,96], and nano-formulations to preserve colour, texture, and quality [97,98,99].

The functionality of chitosan can be further enhanced through a range of chemical modifications (e.g., quaternization, carboxymethylation, alkylation, copolymerization, cross-linking, and hydrolysis) and nanotechnology as well as through combinations with various natural additives, such as essential oils and plant extracts, as reviewed by Yu et al. [100] and Fatima et al. [73]. These innovations have been shown to enhance the solubility, bioavailability, and antimicrobial efficacy of chitosan [73,100]. The utilisation of encapsulation techniques enhances the delivery and stability of chitosan in food and healthcare applications [101,102].

The utilisation of chitosan in various fields also offers environmental and safety benefits. As a natural product delivered from crustacean waste, fungi, and insects, chitosan has been shown to support resource recycling and sustainability [103,104,105]. It is classified as “Generally Recognised as Safe” (GARS) substance by the FDA (GRAS no. GRN 170), the EU (Regulation no. 749/2012), and China (National Standards GB 29941–2013), though caution is needed for individuals with shellfish allergies [106].

## 3. Antimicrobial Mechanism of Chitosan

The most widely accepted theory of the primary antimicrobial mechanism of chitosan, is that its positively charged amino groups of chitosan bind to negatively charged bacterial membrane, disrupting membrane integrity, forming pores, and causing cell leakage and death [107,108,109,110,111]. Chitosan also potentially enters cells, binds to DNA, and interferes with mRNA synthesis and protein production [112].

Further antimicrobial actions of chitosan on bacterial cells include disruption of membranes, inhibition of nutrient transport, chelation of metal ions, and reduction in aerobic bacteria via an oxygen barrier [46,112,113]. The antimicrobial action of chitosan is also influenced by its ability to affect the cell membrane, intracellular nucleic acids, surface proteins, and lipopolysaccharides of microorganisms [114]. In fungi chitosan inhibits sporulation, spore germination, and enzymatic activity within hyphae [105]. It has been established that the antimicrobial activity of chitosan is cell surface-dependent [27,108]. The way in which cationic interaction occurs between chitosan molecules and microbes depends on the type of microbe and the composition of its cell wall [108]. Thus, understanding the outer membrane of microbes is a key to designing effective antimicrobial chitosan structures.

Interaction sites between chitosan and Gram-negative bacteria are associated with electrostatic interactions involving the negative charges of lipopolysaccharide LPS [108], resulting in changes in permeability. Helander et al. [108] compared the antibacterial activity of a normal strain of *Salmonella* Typhimurium with anionic LPS with that of an abnormally cationic LPS mutant strain. Both the normal and mutant *S.* Typhimurium strains in the stationary phase were resistant to chitosan at concentrations of up to 20,000 ppm. However, when the bacteria were grown to the middle of the logarithmic phase, the mutant strain with a high cation content retained viability, whereas the viability of the normal strain decreased by 3 log_10_ CFU/mL [108]. As the outer membrane acts as an effective permeability barrier to macromolecules, the macromolecule chitosan is unable to cross the outer membrane of Gram-negative bacteria [115]. However, Helander et al. [108] demonstrated membrane damage in Gram-negative bacteria treated with chitosan, suggesting that it binds to the outer membrane, leading to a loss of barrier function.

The antibacterial efficacy of chitosan is influenced by its origin and its physicochemical properties, as Nasaj et al. [116] demonstrated in their review. Beyond concentration, degree of deacetylation, temperature, salinity, food surface interactions, microbial type, origin of chitosan, presence of divalent cations, molecular weight, and pH have been identified as the most significant key influencing factors in its action [58,61,83,117]. Acidic conditions/pH enhance the charge and effectiveness of chitosan [116]. A low molecular weight results in better penetration into microbial cells and targeting of internal components, while a high molecular weight is more effective as a surface barrier and membrane disruptor [111,112]. Variations in these properties can lead to inconsistent antibacterial performance, necessitating careful formulation to ensure consistent antibacterial effects in different food matrices.

### 3.1. Antimicrobial Activity of Chitosan Against Listeria monocytogenes in Foods

Chitosan exhibits multiple mechanisms of action against *Listeria monocytogenes*, making it an effective agent for food preservation.

#### 3.1.1. Mechanisms of Antimicrobial Activity of Chitosan Against *Listeria monocytogenes*

**Figure 1:**
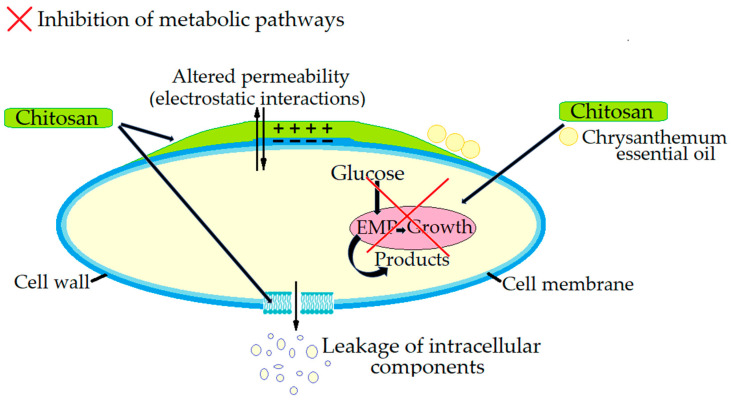
The antimicrobial mechanism of action of chitosan against *Listeria monocytogenes*.

The identified key mechanisms of the antimicrobial action of chitosan against *Listeria monocytogenes* are the following (Figure 1):

- Cell membrane disruption: Chitosan has been demonstrated to disrupt the cell membrane of *L. monocytogenes*, leading to increased cell permeability and the leakage of intracellular contents including ATP, nucleic acids and proteins resulting in cell death [118,119,120].
- Inhibition of metabolic pathways: It has been observed that combining chitosan with chrysanthemum essential oil, can inhibit key metabolic pathways, such as the Embden–Meyerhof–Parnas (EMP) pathway in *L. monocytogenes*, further reducing its viability [121].

#### 3.1.2. Factors Affecting the Antimicrobial Activity of Chitosan Against *Listeria monocytogenes*

Several factors affecting the antimicrobial effect of chitosan against *Listeria monocytogenes* have been identified. These are as follows:

- Molecular weight (MW): As demonstrated by Benabbou et al. [118], the minimum inhibitory concentrations (MICs) of chitosan against *L. monocytogenes* were found to depend on its molecular weight, with lower molecular weight chitosan showing higher MIC values. Similarly, Seo et al. [122] showed that chitosan with molecular weights (MWs) ranging from 104 to 201 kDa exhibited relatively greater antimicrobial activity against *L. monocytogenes* compared to higher molecular weight chitosan. This indicates the importance of molecular weight in its effectiveness as an antimicrobial agent. Lower molecular weight (MW) chitosan (2 kDa) affects cell permeability and growth, while medium- (20 kDa) and high-MW (100 kDa) chitosan may form a barrier on the cell surface, preventing nutrient entry [118]. It is evident that this variability requires careful selection and optimisation of chitosan formulations for specific applications.
- Temperature [123]: Recent studies have demonstrated that chitosan is more effective in combating *L. monocytogenes* at lower temperatures, making it a suitable candidate for food preservation applications [124].
- Concentration: Higher concentrations of chitosan generally result in increased antimicrobial effectiveness [124]. An elevated level of chitosan generally increases its antimicrobial action, regardless of MW [122]. In order to guarantee the stability of the antimicrobial effect, it is essential to determine the appropriate concentration of chitosan for a variety of food products and storage conditions.
- pH levels: The antimicrobial activity of chitosan is pH-dependent, showing higher effectiveness at lower pH levels (e.g., pH 4.5) [118,125]. Other studies indicated better activity against *L. monocytogenes* at slightly higher pH levels (pH 6.2) [117] and at values closer to its pKa (6.2–6.7) [124,126]. Therefore, defining and maintaining optimal pH conditions is crucial for its efficacy.
- Formulation and application: Chitosan films and coatings have been used to inhibit *L. monocytogenes* in various food products [26,127,128,129,130,131]. Films prepared with chitosan of different viscosities showed different levels of effectiveness, with lower viscosity chitosan being more effective at higher bacterial concentrations [132]. Additionally, it was also proved that combining chitosan coatings with essential oils or other antimicrobial agents has the potential to further enhance the antimicrobial effect [133,134].
- Combination with other antimicrobials: Combining chitosan with other antimicrobial agents, such as organic acids, has been proven to enhance its effectiveness. For instance, the combination of chitosan and acetic acid significantly reduced the level of *L. monocytogenes* in ready-to-eat shrimp [135]. The study of Benabbou et al. [118] has demonstrated that incorporating antimicrobial agents such as Divergicin M35, within chitosan films can effectively inhibit the growth of *L. monocytogenes* in food matrices, thereby highlighting the efficacy of utilising chitosan-based films for the control of this pathogen. Chitosan can also be combined with nisin and essential oils to enhance its antibacterial efficacy. These combinations frequently demonstrate additive or synergistic effects, leading to more effective inhibition of *L. monocytogenes* [118,121,136].
- Biofilm inhibition: The study of Orgaz et al. [137] has highlighted the efficacy of chitosan in eliminating both planktonic cells and mature *L. monocytogenes* biofilms, making it a versatile agent in food safety applications. The inhibitory effect of chitosan nanoparticles, particularly when combined with other agents such as DNase I, has been demonstrated in the inhibition of biofilm formation and the disruption of preformed biofilms on food contact surfaces. This was achieved by reducing cell motility and slime production and by causing physical damage to the biofilm structure [120].
- Controlled release of antibacterials: The application of chitosan coatings and films has been found to facilitate the controlled release of antimicrobial agents, thereby ensuring their stability and prolonging their antibacterial activity over time. This is a particularly useful application in the field of food packaging, with the purpose of extending shelf life and ensuring food safety [119,121,138]. Chitosan-stabilised liposomes have been shown to encapsulate antibacterial peptides, which are released in response to bacterial toxins. This targeted release mechanism enhances the antimicrobial effect against *L. monocytogenes* specifically [138]. Chitosan nanoparticles loaded with bacteriocin showed increased antibacterial activity against *L. monocytogenes*, suggesting their potential as effective antibacterial agents in food preservation [139].

#### 3.1.3. Antimicrobial Activity of Chitosan Against *Listeria monocytogenes* in Food Applications

Chitosan has demonstrated significant antimicrobial properties against *Listeria monocytogenes* in various food applications, including meat products, such as pork loins and fishery products like cold-smoked salmon [124,129,130,140,141]. Chitosan films and blends have been recognised as a natural alternative to chemically synthesised antimicrobial polymers for maintaining the quality and microbiological safety of food products [142,143]. When incorporated into low-density polyethylene (LDPE) films, chitosan not only inhibited microbial growth and extended the shelf life of red meat, but also preserved its colour [144]. Chitosan coatings on vacuum-packed fresh pork significantly reduced *L. monocytogenes* counts and improved shelf life without affecting sensory properties [145]. Similarly, chitosan coatings on ready-to-eat roast beef reduced *L. monocytogenes* counts by 2–3 log_10_ CFU/g over 28 days [129]. Chitosan films enriched with essential oils (EOs), such as oregano oil, have shown enhanced antimicrobial activity. These films reduced *L. monocytogenes* by 3.6 to 4 logs on processed meat, demonstrating their potential as active biodegradable packaging materials [146]. Chitosan coatings containing sodium lactate, sodium diacetate, and potassium sorbate achieved significant log reductions in *L. monocytogenes* on cold-smoked salmon [130].

Chitosan can also be used effectively in dairy products. Chitosan-coated nisin-silica liposomes have demonstrated sustained antibacterial activity against *L. monocytogenes* in cheese, without compromising its sensory characteristics, indicating their potential for cheese preservation [136]. In the research study of Sandoval et al. [147] the incorporation of chitosan-grafted lactic acid packaging into the packaging of fresh cheese resulted in a significant extension of the shelf life of the cheese, with the growth of *L. monocytogenes* being effectively inhibited during a 14-day storage period at a temperature of 4 °C. Further food applications are shown in Table 1.

In summary, it can be concluded that chitosan exhibits remarkable antimicrobial properties against *L. monocytogenes*, particularly when applied in a combination with other antimicrobials or in specific formulations, such as films and coatings. Consequently, it possesses versatile potential for enhancing food safety. However, it should be noted that a common issue for the publications included in this review is that the authors failed to perform presence–absence tests during the experiments, conducting only colony counting methods instead. According to the Commission Regulation (EC) No 2073/2005 [160], there are two different limits for *L. monocytogenes* in “ready-to-eat” food products. If the product does not support the growth of *L. monocytogenes*, counts below 100 CFU/g or 100 CFU/mL are permitted, and the colony counting method can be used. However, if the food product supports the growth of *L. monocytogenes*, zero tolerance applies, i.e., a presence/absence test must be performed. In a presence/absence test, *Listeria monocytogenes* must be absent (undetectable) in five sample units of 25 g each, i.e., it must not be detectable in 125 g of product. Therefore, during the experiments, if the cell count is below the detection limit, it cannot be proven that the product is free of the pathogen and safe to consume. It can only be concluded that the pathogen count is below the detection limit; however, this does not guarantee that 25 g or 25 mL of food are free of this pathogen. Therefore, the reduction in *L. monocytogenes* to undetectable levels [154] or complete reduction in *L. monocytogenes* [156] does not necessarily mean a safe, pathogen-free food.

### 3.2. Antimicrobial Activity of Chitosan Against Staphylococcus aureus in Foods

Chitosan exhibits significant antimicrobial activity against *Staphylococcus aureus*, making it a promising agent for combating this pathogen.

#### 3.2.1. Mechanisms of Antimicrobial Activity of Chitosan Against *Staphylococcus aureus*

**Figure 2:**
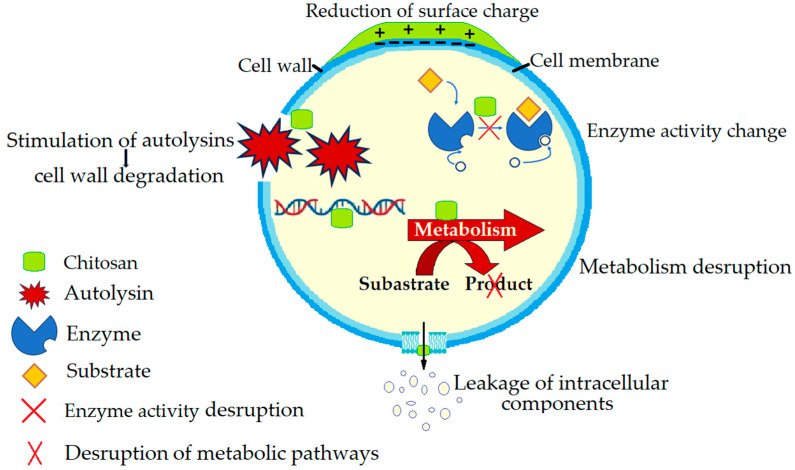
The antimicrobial mechanism of action of chitosan against *Staphylococcus aureus*.

The mechanisms by which chitosan inhibits the growth of *S. aureus* in food products are complex and include the following (Figure 2):

- Cell wall and membrane disruption: Chitosan interacts with and disrupts the bacterial cell wall and cytoplasmic membrane of *S. aureus*, causing structural disorganisation and increased cell permeability, leading to the leakage of cellular contents into the environment and bacterial death [161,162].
- Stimulation of autolysins: Chitosan can stimulate the degradation of bacterial cell walls by promoting the activity of bacterial autolysins in *S. aureus*. This mechanism enhances the breakdown of the cell wall, contributing to the antibacterial effect [163].
- Change in metabolism: Chitosan can disrupt the normal metabolism of *S. aureus*, further inhibiting bacterial growth [164].
- Enzyme activity disruption: Chitosan-grafted derivatives can disrupt the normal metabolism of *S. aureus* by affecting the activity of cellular antioxidant enzymes and intracellular enzymes, leading to bacterial cell damage and death [164].
- Reduction in surface charge: Chitosan interacts with the anionic cell wall of *S. aureus*, reducing the surface charge and thereby inhibiting bacterial adhesion and colonisation. This interaction is crucial for the antibacterial activity of chitosan, as demonstrated by the reduced adsorption of *S. aureus* onto chitosan when the surface charge is neutralised [165].
- Inhibition of DNA synthesis: Chitosan derivatives can penetrate bacterial cells through damaged membranes and inhibit DNA synthesis in *S. aureus*, further preventing bacterial replication and growth [166].
- Protonation of amino groups: The antibacterial action of chitosan-based nanofibers (CNFs) is attributed to the protonation of their amino groups. This protonation enhances the bactericidal activity of chitosan, making it effective against various strains, including *S. aureus* [167].

#### 3.2.2. Factors Affecting the Antimicrobial Activity of Chitosan Against *Staphylococcus aureus*

The following factors have been demonstrated to influence the antimicrobial effect of chitosan against *S. aureus* in food:

- Biofilm inhibition: Chitosan also interferes with biofilm formation, making it effective against *S. aureus* [164,168]. The antimicrobial activity of chitosan against *S. aureus* has been demonstrated in both planktonic and sessile settings, including significant antibiofilm activity [137,164,168].
- Molecular weight: The antibacterial effect of chitosan on *S. aureus* is influenced by the molecular weight of chitosan, with 50 kDa molecular weight chitosan exhibiting higher antibacterial activity against *S. aureus* compared to 5 kDa chitosan [169]. However, chitosan with molecular weights ranging from 104 to 201 kDa showed greater antimicrobial activity against *S. aureus* compared to higher molecular weights [122]. Higher molecular weight chitosan (below 300 kDa) enhanced the antimicrobial effect on *S. aureus* [170].
- Concentration: Zheng and Zhu [170] demonstrated that increasing the concentration of chitosan resulted in a stronger antimicrobial effect. When the initial concentration was elevated to 1.0%, the inhibition rate for *S. aureus* was observed to reach 100%. Chitosan exhibits a pronounced antimicrobial effect against *S. aureus*, with higher concentrations leading to greater inhibition. A 1% concentration of chitosan completely inhibited *S. aureus* in cheese after the first day of storage, while a 0.5% concentration achieved complete inhibition by the fifth day of storage at 4 °C [171]. Although higher concentrations generally increase antibacterial activity, Ardila et al. [126] suggest that there is a critical point beyond which the effect may also decrease in *S. aureus*. This can be attributed to the presence of proteins that act as nutrients for bacteria.
- Presence of food components: The presence of certain food components can affect the antibacterial activity of chitosan on *S. aureus*. For example, acetic acid, lactic acid, and citric acid enhanced the inhibitory effect of chitosan, while NaCl slightly reduced it [172].
- Temperature and ionic strength: Higher temperatures and an appropriate ionic strength promote the antibacterial activity of chitosan against *S. aureus*. These factors increase its effectiveness by enhancing the attachment of the cells to chitosan [126]. Refrigeration enhanced its antibacterial activity compared to ambient temperatures [173].
- Combination with other antimicrobials: Chitosan films, especially when combined with other bioactive compounds like nisin or garlic oil, show enhanced activity against *S. aureus* [174,175]. Incorporating essential oils (EOs), such as clove oil, into chitosan films can significantly boosts their antimicrobial properties, with notable inhibition against *S. aureus* [176]. Combining chitosan with silver nanoparticles can significantly enhance its antimicrobial efficacy [177,178].

#### 3.2.3. Antimicrobial Activity of Chitosan Against *Staphylococcus aureus* in Food Applications

Chitosan can be used as a natural food preservative in various forms, including solutions, films, and coatings, to extend the shelf life of food products by inhibiting the growth of *S. aureus* [33,171,175]. Its incorporation into biodegradable films and packaging materials provides an environmentally friendly solution for ensuring food safety [178]. These films are effective in controlling the microbial growth of *S. aureus* in minimally processed foods such as pears [175]. Chitosan solutions at concentrations ranging from 0.5% to 2% were effective in reducing counts of both *S. aureus* and methicillin-resistant *S. aureus* in frozen and fresh beef. The highest reduction was observed with 2% chitosan, which significantly decreased bacterial counts at refrigeration temperature [173]. Chitosan films incorporated with natural white ginger essential oil (GEO) were effective in inhibiting *S. aureus* growth on fruits such as apples and pears. The blend of chitosan and GEO suppressed microbial growth and reduced fruit weight loss [179]. Chitosan-based coatings containing eugenol and oregano essential oil demonstrated antimicrobial activity against *S. aureus*. These coatings were effective in preserving fresh cheese by reducing colony-forming units of *S. aureus* during storage [180]. Chitosan has also been found to effectively inhibit the growth of *S. aureus* in sushi rice [181]. Other applications can be found in Table 2.

In summary, chitosan demonstrates significant antimicrobial activity against *S. aureus* in foods. Its effectiveness can be further enhanced by environmental factors and synergistic combinations with other antimicrobial agents, offering a versatile solution for controlling *S. aureus* and improving food safety.

### 3.3. Antimicrobial Activity of Chitosan Against Escherichia coli O157:H7 in Foods

Chitosan exhibited significant antimicrobial activity against *E. coli* O157:H7 through multiple mechanisms, primarily targeting the bacterial cell membrane and cell wall.

#### 3.3.1. Mechanisms of Antimicrobial Activity of Chitosan Against *E. coli* O157:H7

**Figure 3:**
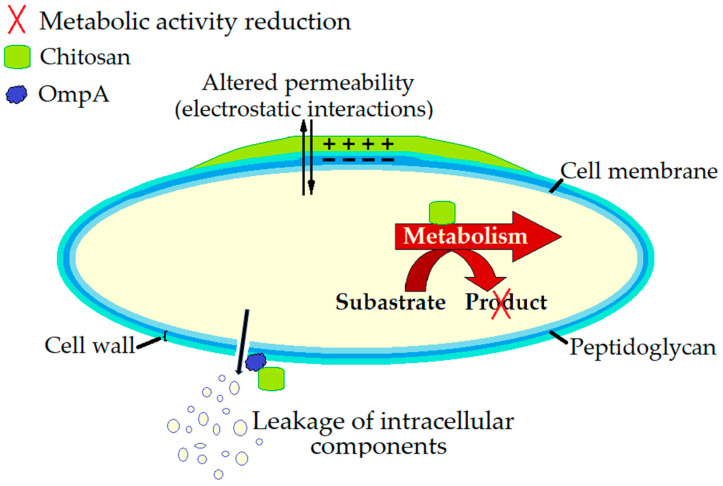
The antimicrobial mechanism of action of chitosan against *E. coli* O157:H7.

The key mechanisms of the antimicrobial action of chitosan against *E. coli* O157:H7 are (Figure 3):

- Cell membrane disruption: Chitosan disrupts the integrity of the bacterial cell membrane of *E. coli* O157:H7 resulting in the release of DNA and other cellular components, leading to the leakage of intracellular contents and eventual cell death [186,187]. As evidenced by Gu et al. [186], chitosan treatment resulted in the destruction of various macromolecular components, including fatty acids, proteins, peptidoglycans, glycoside rings, and polysaccharides in *E. coli* O157:H7 cells. The cell membrane exhibited local displacement and reduced thickness, and large molecules adhered to the cell surface, resulting in the formation of holes and subsequent leakage of intracellular contents, ultimately leading to cell death. Jeon et al. [187] proved that the binding of chitosan to the outer membrane protein OmpA of *E. coli* O157:H7 is critical for its bactericidal effect, causing membrane disorganisation and leakage.
- Modification of chitosan changes in metabolic activity: A novel water-soluble chitosan derivative, arginine-functionalized chitosan, showed dose-dependent inhibition of *E. coli* O157:H7 with greater inhibition at higher concentration, reducing both pathogen numbers and metabolic activity [188]. Chitosan-arginine, which is soluble and active at neutral and basic pH showed antimicrobial effect against *E. coli* O157:H7, reducing the viability and metabolic activity of the cells held in stationary phase [189].

#### 3.3.2. Factors Affecting the Antimicrobial Activity of Chitosan Against *E. coli* O157:H7

The factors affecting the antimicrobial effect of chitosan against *E. coli* O157:H7 have been identified as follows:

- pH level: The effectiveness of chitosan against *E. coli* O157:H7 was influenced by the pH level of the environment, with higher activity observed at a pH level of 6.2 compared to pH 5.0 [117].
- Temperature: The antimicrobial activity of chitosan is influenced by the temperature, with higher activity observed at refrigeration temperatures [117].
- Concentration: The antibacterial activity of chitosan is dose-dependent and increases with its concentration. For instance, chitosan concentrations of 0.1% and 0.7% of chitosan were effective against *E. coli* O157:H7, with higher concentrations showing greater bactericidal effects [74,190].
- Molecular weight: The molecular weight of chitosan plays an important role in its antimicrobial activity. Seo et al. [122] demonstrated that intermediate molecular weight chitosan was more effective compared to lower or higher molecular weight chitosan at inhibiting the growth of *E. coli* O157:H7, particularly at a concentration of 0.1%.
- Combination with other antimicrobials: Combining chitosan with other antimicrobial agents, such as the extracellular metabolites of *Pediococcus pentosaceus* or gum arabic, resulted in an additive effect, significantly reducing *E. coli* O157:H7 contamination on food surfaces [190,191]. Combining chitosan with essential oils, bacteriocins, or citrus extracts, also enhances its effectiveness against *E. coli* O157:H7 [192,193,194]. A combination of citrus extract and chitosan showed an additive inhibitory effect against this pathogenic bacterium [192]. Chitosan combined with essential oils, such as clove and thyme, showed stronger antibacterial activity against *E. coli* O157:H7 than chitosan alone [193]. Similarly, chitosan-based coatings containing nano-emulsions of essential oils and gamma irradiation significantly increased the radiosensitisation of *E. coli* O157:H7 [194].

#### 3.3.3. Antimicrobial Activity of Chitosan Against *E. coli* O157:H7 in Food Applications

Chitosan has demonstrated antimicrobial activity against *E. coli* O157:7 in various foods [188,190,195]. It has been shown to inhibit the growth of *E. coli* O157:H7 in broccoli, resulting in a significant reduction in total *E. coli* counts, and demonstrating its potential for controlling microbial contamination [195]. The chitosan coatings used on broccoli also improved its sensory quality by inhibiting yellowing and the opening of florets. A combination of chitosan and the extracellular metabolites of *Pediococcus pentosaceus* showed an additive effect, significantly reducing the number of *E. coli* O157:H7 on the surface of cantaloupe [190]. Chitosan-arginine at higher concentrations (up to 500 mg/L) significantly reduced the numbers and metabolic activity of *E. coli* O157 in chicken juice in a dose-dependent manner [189]. Chitosan at a concentration of 5% (*w*/*v*) was found to effectively reduce contamination in local Iraqi cheese products, although it did not completely eliminate *E. coli* O157:H7 [191].

Chitosan-based films and edible coatings can be used in food packaging to maintain product shelf life and freshness, enhance the microbial foods safety by minimising the risk of *E. coli* O157:H7 infection, and control microbial growth during storage [188]. These coatings can incorporate various compounds, including lytic bacteriophages, which have been shown to be effective in reducing *E. coli* O157:H7 levels on food surfaces, such as tomatoes [196]. The incorporation of essential oils into chitosan films can further enhance their antimicrobial properties [193]. Chitosan films containing lactoferrin and lysozyme have demonstrated significant antimicrobial activity against *E. coli* O157:H7. The combination of these agents in chitosan films results in a notable reduction in bacterial growth, making it a potent food preservation strategy [197]. Other potential food applications are detailed in Table 3. Using chitosan in combination with other antimicrobial agents usually provides a more robust defence against *E. coli* O157:H7, although complete elimination of the pathogen may not always be achieved [189,190,192] necessitating its use as part of a broader food safety strategy. The study of Kiskó et al. [74] demonstrated that supplementation of chitosan in apple juice retarded the deterioration of the product caused by yeasts; however, it has also been shown to enhance the survival of *E. coli* O157:H7. Although plate counting showed that the number of the pathogen was below the detection limit, the presence/absence results showed that living pathogen bacteria were still detectable in the juice during storage. The findings of this study indicate that the utilisation of chitosan in the treatment of fruit juices may result in an elevated risk of food poisoning from *E. coli* O157:H7.

In summary, chitosan exhibits good antimicrobial properties against *E. coli* O157:H7 in various food products, particularly when used combined with other antimicrobial agents. Its application in food preservation and packaging can significantly enhance food safety, although it should be considered a protective measure rather than a complete solution for pathogen elimination.

### 3.4. Antimicrobial Activity of Chitosan Against Salmonella in Foods

A mechanism of the antimicrobial effect of chitosan against *Salmonella* spp. has also been identified: the disruption of cell membranes.

#### 3.4.1. Mechanism of Antimicrobial Activity of Chitosan Against *Salmonella*

**Figure 4:**
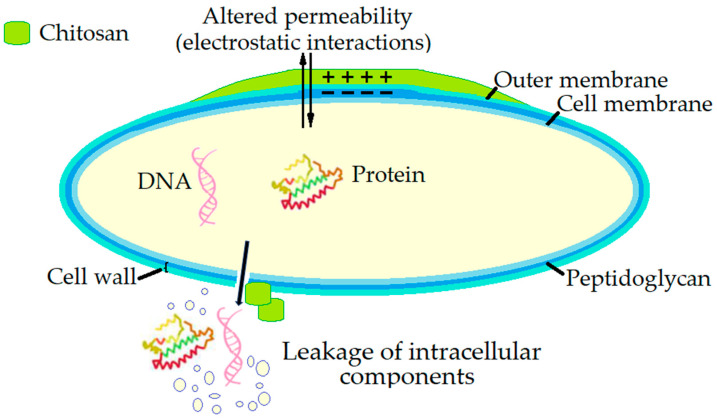
The antimicrobial mechanism of action of chitosan against *Salmonella*.

- The identified mechanism of the antimicrobial activity of chitosan against *Salmonella* is the disruption of cell membranes (Figure 4). Chitosan interacts with the negatively charged bacterial cell wall also in *Salmonella* cells, causing membrane rupture and leakage of intracellular components such as proteins and DNA. This membrane permeabilisation and perforation are evident from the release of these components and the formation of pores observed under transmission electron microscopy (TEM) [167].

#### 3.4.2. Factors Affecting the Antimicrobial Activity of Chitosan Against *Salmonella*

Numerous factors can influence the effectiveness of the activity of chitosan against *Salmonella*, including the following:

- Deacetylation degree and molecular weight: The degree of deacetylation of chitosan influences its effectiveness, with lower acetylation and higher molecular weight chitosan showing better antibacterial activity [203].
- Form of chitosan: The antibacterial activity of chitosan is also enhanced when it is in the form of nanofibers or nanoparticles, which show high efficacy compared to other physical forms. This is likely due to the increased surface area and better interaction with bacterial cells [126,204].
- Source of chitosan: The findings of Ibañez-Peinado et al. [117] demonstrated that the activity of the chitosan derived from insects was less effective against *Salmonella* than its crustacean-derived counterpart.
- Effect of food components: The presence of certain food components can affect the antimicrobial activity of chitosan. While NaCl and sucrose can slightly decrease its inhibitory activity, the addition of acids such as acetic, lactic, and citric acid can enhance the effectiveness of chitosan against bacterial growth, including *Salmonella* [172].
- Temperature: The antimicrobial activity of chitosan decreased at higher storage temperatures. It reduced the growth of *Salmonella* at 4 °C, but increased it at 10 °C [205].
- Antibiofilm activity: Chitosan can inhibit biofilm formation, which is crucial for preventing bacterial colonisation and persistence in food products. Studies have shown that chitosan combined with medicinal leaf extracts of *Mentha piperita* L. and *Plectranthus amboinicus* significantly reduced biofilm formation by *Salmonella* spp. [206]. This combination also enhanced the antimicrobial activity against multidrug-resistant strains of *Salmonella*.
- Combination with other antimicrobials: Combining chitosan with other antimicrobial agents such as nisin, allylisothiocyanate, and essential oils can enhance its effectiveness against *Salmonella* [175,207,208]. Chitosan films incorporated with 1,8-cineole, an active component in essential oils, have been shown to effectively retard the growth of *Salmonella* on food surfaces [209]. Additionally, combining chitosan with bacteriocins from *Carnobacterium maltaromaticum* has demonstrated increased antibacterial efficacy against *Salmonella* in beef [207].

#### 3.4.3. Antimicrobial Activity of Chitosan Against *Salmonella* in Food Applications

Many applications of chitosan in food packaging proved to be effective against *Salmonella*. Chitosan was successfully applied in animal foods such as meat and eggs. Chitosan-coated films (2% chitosan coating) have been shown to reduce trans-shell penetration of *Salmonella* Enteritidis in eggs, thereby decreasing contamination [210]. Beef treated with steam followed by the addition of chitosan and bacteriocins from *Carnobacterium* species resulted in 3 log_10_ (CFU/cm^2^) reduction in *S. enterica* counts during refrigerated storage [207]. The combination of citrus extract and chitosan demonstrated an additive inhibitory effect against *S. enterica*, reducing the population by approximately 2.2 or 5.6 log_10_ CFU/g in vacuum-packed turkey meat on day 21 of storage at 4 and 10 °C [192]. The application of a chitosan-based thymol nano-emulsion coating in ground chicken meat has been shown to reduce cross-contamination of *S. enteritidis* by up to 1.91 log_10_ CFU/g [211]. The use of tea tree oil liposomes/chitosan nanofibres at temperatures of 12 °C and 25 °C for a period of 4 days resulted in an approximately 5 log_10_ CFU/g reduction in Salmonella in chicken meat [212]. In a recent study, the effects of edible chitosan films with either a carvacrol nano-emulsion (1.56%) or a rosemary nano-emulsion (1.56%) on the viability of *S.* Typhimurium in minced meat samples were investigated. It was observed that the chitosan film with carvacrol nano-emulsion (1.56%) was able to reduce the levels of *S*. Typhimurium by 2.5 log_10_ CFU/g in inoculated minced meat samples. In contrast, the chitosan film with rosemary nano-emulsion (1.56%) continued to reduce the counts to 2 and 3 log_10_ CFU/g, respectively [158].

*Salmonella* counts on fruit and vegetables can also be successfully reduced using chitosan. Chitosan coatings on cantaloupes, especially when combined with allylisothiocyanate and nisin, significantly reduced *Salmonella* populations [208]. Lee et al. [213] observed a 2.51 log_10_ CFU/mL reduction in Salmonella Typhimurium after 48 h of incubation in orange juice, while Kiskó et al. [74] demonstrated that the survival of *S.* Typhimurium was unaffected by chitosan at either 4 °C or 25 °C in unpasteurised apple juice. Won et al. [214] investigated the efficacy of coating cherry tomatoes with a solution comprising chitosan colloids and grapefruit seed extract (GSE) at concentrations of 0.0%, 0.5%, 0.7% and 1.0% (*w*/*w*). Depending on the concentration of GSE, Inactivation of *Salmonella* bacteria was achieved, with reductions ranging from 1.0 ± 0.3 log_10_ CFU/cherry tomato to 2.0 ± 0.3 log_10_ CFU/cherry tomato. Chitosan nanoparticle solutions also have a great potential as a disinfectant wash for fresh vegetables. It has been demonstrated that the application of a chitosan nanoparticles washing solution is effective in eliminating of more than 1 log_10_ of inoculated populations of *Salmonella* Typhimurium on lettuce [215]. It was also demonstrated that applying a chitosan coating on asparagus spears resulted in a significant log reduction of 1.5 log_10_ CFU/g of *Salmonella* spp. [216]. Other potential applications are shown in Table 4.

In conclusion, chitosan demonstrates promising antimicrobial effects against *Salmonella* in various food applications. Its ability to form films, nanoparticles, and coatings, combined with its biocompatibility and biodegradability, makes it a valuable tool in combatting *Salmonella* contamination in food products. Incorporating essential oils and other antimicrobial agents further enhances its efficacy, providing a promising approach to ensuring food safety.

## 4. Benefits Associated with the Application of Chitosan

The use of chitosan in food products presents numerous benefits, making it a valuable tool in food preservation and safety. It has been shown to have a broad antimicrobial spectrum, being highly susceptible to a wide variety of pathogenic and spoilage microorganisms, including fungi, Gram-positive and Gram-negative bacteria, making it a versatile antimicrobial agent [217]. This broad-spectrum efficacy can help enhance food safety and reduce the risk of foodborne illnesses. The antimicrobial activity of chitosan can be optimised by adjusting its molecular weight and degree of deacetylation or by combining it with other antimicrobial agents. These modifications can enhance its solubility and effectiveness.

The versatile applicability of chitosan (which can be used in a variety of structures, including films, coatings and nanoparticles) enables its flexible application in preservation of foodstuffs [26,218]. This versatility allows for tailored applications depending on the type of food product and the desired preservation method. The utilisation of chitosan possesses the potential to maintain the quality of food products by preventing spoilage and extending shelf life. This can lead to a reduced amount of food waste and an improved economic efficiency. The ability of chitosan to regulate microbial growth contributes to the preservation of sensory and nutritional quality of food products as well, thereby ensuring their appeal to consumers over time.

Chitosan can enhance the effectiveness of other preservatives when used in combination. Its antimicrobial activity can be synergistically increased when combined with a variety of other substances, including but not limited to, organic acids, essential oils, bacteriocins, plant extracts, graphene, titanium dioxide, and zinc oxide. This provides a more robust preservation strategy which is effective not only against *Listeria monocytogenes*, *Staphylococcus aureus*, *E. coli* O157:H7, and *Salmonella* species but other pathogens as well [219,220,221,222,223].

The non-toxic and biodegradable nature of chitosan renders it an environmentally friendly alternative to synthetic preservatives, thus appealing to consumers seeking clean-label products. Chitosan has been extensively investigated for its potential use in the production of new edible films and multifunctional formulations for various food applications, making it a promising substitute for synthetic plastic polymers [224]. Its incorporation into food packaging has the potential to reduce the environmental impact of plastic waste [26,143,218].

When utilising chitosan as a coating, it is necessary to consider its impact on the sensory characteristics of food products. Some research showed that the application of chitosan-coated nisin–silica liposomes did not result in any alteration of the sensory properties of cheese, indicating potential for use in food preservation without compromising quality [136,225,226].

Chitosan has been demonstrated to have a number of health benefits, including the capacity to reduce cholesterol levels and the potential to exhibit anti-inflammatory properties. The incorporation of chitosan into food products has the potential to enhance not only safety but also the overall health benefits of the food [38,227].

Whilst it is essential to consider the primary production costs associated with the production of chitosan, its ability to extend shelf life and reduce spoilage can lead to overall cost savings in food production and distribution.

## 5. Challenges Associated with the Application of Chitosan

Despite the numerous advantages attributed to chitosan, weaknesses have also been identified. There are still challenges and research gaps regarding the antimicrobial action of chitosan. The exact mechanism remains only partially understood. More targeted research is needed to optimise the performance of chitosan and to understand its full antimicrobial spectrum.

In food preservation, chitosan may negatively affect taste and texture when used in higher concentrations [146,149,228,229]. Challenges in the applications of chitosan include low solubility in neutral/alkaline pH [111,112]. This property may limit its application in neutral or alkaline food products. This solubility issue has the potential to compromise its effectiveness in a range of food matrices where pH levels may fluctuate.

Although chitosan is widely considered to have low potential to induce resistance, prolonged use could still lead to the development of resistant strains of pathogenic, spoilage, or even useful bacteria, such as starter cultures. These non-pathogenic bacteria then have the potential to transfer the resistance genes to pathogenic bacteria via horizontal gene transfer. Research reports have demonstrated the presence of chitosan-resistant *Staphylococcus aureus* and fungi, supporting the hypothesis of induced resistance against chitosan [46,230].

The antimicrobial activity of chitosan is influenced by a variety of factors including its molecular weight and degree of deacetylation [123]. In complex food systems, these properties may be altered due to interactions with other food components; therefore, complex food matrices potentially may reduce its effectiveness, limiting its practical application. This highlights the importance of considering the specific food matrix in its use. Combined strategies are being explored to overcome these issues [58]. It has also been demonstrated that chitosan may exhibit potential adverse nutritional impacts. In high doses, it can reduce the levels of vitamin C in food due to its flocculation effect, indicating the need for lower, balanced doses [231]. Chitosan and its films often have weaker antibacterial effects, less thermal and mechanical stability, and poorer barrier properties against gases and moisture when compared to conventional antibacterial agents and plastics [100,232,233,234,235]. However, these properties are essential for preserving food quality improving the barrier characteristics of chitosan-based packaging is therefore necessary.

Furthermore, the combination of chitosan with other materials, such as gelatine, has been demonstrated to enhance its performance; however, these composites frequently still lack the water resistance and mechanical strength found in synthetic plastics [236]. These weaknesses can restrict their application in antibacterial food packaging materials to a certain degree. Additionally, the study of No et al. [237] found that chitosan solutions stored at 25 °C exhibited reduced antibacterial activity compared to those stored at 4 °C, suggesting a decrease in efficacy with prolonged storage time. Its antioxidant and bacteriostatic capacity are limited by chemical inertness and strong internal hydrogen bonding [238,239]. Its effectiveness varies due to influences from environmental and formulation factors [240,241]. Inconsistent properties of chitosan sources and a lack of standardisation in the extraction and application methods are barriers to consistent performance.

Compliance with labelling regulations for food packaging materials that contain antimicrobial agents is necessary. It is imperative that products are clearly and accurately labelled to inform consumers of the presence of chitosan and its intended benefits.

Chitosan films containing specific antimicrobial compositions achieved significant reductions in pathogenic bacteria including *Listeria monocytogenes*, *Staphylococcus aureus*, *E. coli* O157:H7, and *Salmonella* species. This emphasises the importance of selecting appropriate antimicrobial agents for incorporation into chitosan-based packaging. The utilisation of chitosan-based films, particularly in instances where nanoparticles or other bioactive compounds are incorporated and chitosan-stabilised liposomes encapsulating antibacterial substances has been demonstrated to be an effective approach for controlling foodborne pathogens. However, when implemented in food processing environments, their application poses a regulatory challenge. The navigation of the regulatory framework can be a time-consuming and costly process.

Chitosan is composed of a variety of structurally diverse chemical entities, each of which may have distinct biodistribution, biodegradation, and toxicological profiles [51]. A comprehensive evaluation is necessary to determine their potential toxicity and environmental impact [138,242,243]. To ensure safe food applications, it is necessary to understand the cytotoxicity.

It is also essential to define the optimal concentration and release rate of chitosan in packaging materials to ensure the effective preservation of its antimicrobial properties against foodborne pathogens throughout the shelf life of the product.

Furthermore, the stability of chitosan in food products must be considered, as it may degrade over time or lose its antimicrobial properties due to environmental factors such as temperature and moisture [244,245,246,247]. It is imperative that consistent quality and performance of chitosan and modified chitosan compounds at a larger scale are ensured for successful implementation.

Consumer acceptance can also affect the applicability of chitosan packaging. It is noteworthy that while chitosan is a natural biopolymer, consumer acceptance of chitosan-based packaging may vary. The appearance and texture of chitosan-based packaging may differ from traditional packaging materials, which may have a negative effect on consumer preferences. It is important to ensure that the packaging is visually attractive and does not affect the sensory qualities of the food. Therefore, it is essential to educate consumers about the benefits and safety of chitosan as an antimicrobial agent to encourage its widespread adoption.

In addition to the aforementioned challenges, it has been demonstrated that the production of chitosan-based packaging materials may be more expensive than conventional packaging options. Balancing the cost of production with the benefits of enhanced food safety is a challenge for manufacturers.

## 6. Research Directions

Recent research trends have placed significant emphasis on the in vivo antimicrobial activity of chitosan and its derivatives, such as carboxymethyl chitosan and N,N,N-trimethyl chitosan, as well as on the antimicrobial activity of their micro- and nanoparticle forms. In addition to increasing food safety, they enhance plant protection, the effectiveness of animal diseases treatments and wound healing applications [114,248,249]. Recent studies have focused on enhancing the antimicrobial effect of chitosan through synergistic approaches combining chitosan with other antimicrobials [141,250,251,252]. Further discussion is required on the accepted and potential mechanisms of using chitosan and its derivatives in more detail to improve our understanding of their antimicrobial properties [249].

Future research directions for the utilisation of chitosan as a natural antimicrobial agent within the food industry could concentrate on the further development of chitosan-based nanosystems and their applications to enhance antimicrobial potential. Molecularly engineered nanomaterials, such as acid-transforming chitosan and chitosan with fragment DNA polyplexes, have demonstrated their potential as effective and safe antimicrobial agents against *Salmonella* Typhimurium [253], suggesting future advancements in the development of molecularly engineered nanomaterials as efficient and safe antimicrobial agents for ensuring food safety.

Other areas for research could include the development of chitosan derivatives and conjugates with novel polymers and nanoparticles, which exhibit superior antimicrobial properties. Another research direction could involve the development of new types of films, such as chitosan–gelatine-based films with a mixture of chitosan and gelatine, or further biodegradable chitosan–starch films. The combination of these films with varying concentrations of natural antimicrobials holds promise for their utilisation as temperature-sensitive active packaging films [220,254]. There is a potential for further development of the practical application of chitosan and its combinations in innovative ways, such as bio-inks for 3D and 4D printing [255]. Further research directions could include the development of intelligent films (smart films) based on chitosan [243,256,257,258].

## 7. Conclusions

Chitosan is a natural, non-toxic, biodegradable, commercially available biopolymer with notable bioactivity, including antimicrobial and antioxidant properties, and biodegradability. It, therefore, has considerable potential in enhancing the quality and safety of food products. Research has shown that the natural origin of this “green” biopolymer aligns well with current trends towards healthier and more sustainable food options.

The versatility of chitosan comes from its modifiability, which has attracted attention in the food industry. It can be used as a natural preservative or as edible packaging with antimicrobial properties. As a future alternative to non-biodegradable plastics, it improves food quality and safety.

This review article discusses and summarises the current state of research on the application of chitosan in food preservation, with a particular focus on its use against four major food pathogens—*Listeria monocytogenes*, *Staphylococcus aureus*, *Salmonella* serotypes, and *Escherichia coli* O157:H7—with the aim of helping readers understand the importance of chitosan in food preservation and food safety, and inspiring future developments in this field.

The mechanism of antibacterial action of chitosan involves membrane integrity loss, enzyme inhibition, and several other mechanisms. Together, these ensure the effectiveness of chitosan as a biopreservative agent against pathogenic bacteria in food. Understanding the effects of these factors is essential for optimising chitosan use in food preservation.

As demonstrated in this review article, chitosan and its derivatives show bactericidal activity against *Listeria monocytogenes*, *Staphylococcus aureus*, *Salmonella* spp. and *Escherichia coli* O157:H7. Research have found that using chitosan and its derivatives in or on food can make food last longer and protect against these harmful bacteria. Its applications may preserve or sometimes improve food properties while effectively reducing pathogen contamination. Combining chitosan with other natural substances makes it even more effective against these food-borne pathogens.

Significant advances have clarified the benefits of chitosan for food safety and preservation, but there are still practical challenges and research gaps. Further studies are essential to achieve a comprehensive understanding of food preservation by chitosan. In the future, it is necessary to investigate and fully understand the processes behind its antibacterial activity, and methods to maximise its antimicrobial efficacy.

It is important to note that food components significantly influence the antimicrobial activity of chitosan. Therefore, it is essential to examine product matrix effects, suitable concentrations/combinations for food use and investigate the optimal conditions for maximal antimicrobial activity.

Furthermore, emphasis should be placed on monitoring and preventing the possible development of resistance to chitosan during its widespread use as an antimicrobial agent, because bacteria may gradually adapt to its antimicrobial mechanism.

## Figures and Tables

**Table 1:** Antimicrobial effect of different chitosan applications against *Listeria monocytogenes* in laboratory media and foods.

Chitosan Form	Antimicrobial Concentration	Medium/Food Matrix	Observed Antimicrobial Effect	Reference
Chitosan form *Mucor rouxxi* UCP 064	5.0 and 2.5 mg/mL	Yam bean medium	Bactericidal effect on *L. monocytogenes* in a maximum time of 4 h	[148]
Chitosan form *Mucor rouxxi* UCP 064	5 mg/g	Bovine meat pâté at 4 °C	reduction in *L. monocytogenes* by approximately 3 log_10_ CFU/g after 6 days	[149]
Low-molecular-weight chitosan from shrimp shells, (≥75% deacetylated)	0.02 to 2.0 mg/mL	Luria–Bertani broth	Smallest particles (263 nm) resulted in lower minimum inhibitory concentration of 0.04 mg/mL of *L. monocytogenes*; largest particles, (607 nm) resulted in higher minimum inhibitory concentration of 0.03 mg/mL of *L. monocytogenes*	[150]
Low-molecular-weight chitosan	0.1%	Muller–Hinton agar	Suppression of growth of *L. monocytogenes* completely by 0.03% chitosan at or below pH 5.5	[28]
Cellulose casing impregnated with chitosan	2%	Ready-to-eat (RTE) Vienna sausage	Growth of *L. monocytogenes* Scott A was retarded at 4 and 10 °C throughout the storage for 28 and 5 days	[151]
Chitosan film with sodium lactate	2%	Ham steaks at 4 °C	Reduction in *L. monocytogenes* from 2.7 to 1.5 log_10_ CFU/cm^2^ for 10 weeks, 5.3 log lower than in the control	[128]
Chitosan with high hydrostatic pressures	0.1% chitosan + 250 MPa	Lab medium (ACES buffer)	Synergistic inhibition of *L. monocytogenes* up to 1 log reduction	[152]
Chitosan with 460 nm LED illumination	1.0% chitosan + 460 nm LED 1.3 kJ/cm^2^	Fresh-cut melon	1.5–3.5 log_10_/cm^2^ reduction in *L. monocytogenes*	[153]
Chitosan with 460 nm LED illumination	1.0% chitosan + 460 nm LED illumination at 0.6–0.8 kJ/cm^2^	Fresh-cut melon	Inoculation level of 10^4–5^ CFU/cm^2^ *L. monocytogenes* reduced to undetectable levels	[154]
Chitosan coating	1% and 2%	Vacuum-packed pork loins at 4 °C	reduction in *L. monocytogenes* by Approximately 1.5 to 2 log_10_ CFU/g after 7 days; up to 28 days of inhibition at 2% concentration	[145]
Chitosan-ZnO nanocomposite	1% ZnO	White brined cheese at 4 °C and 10 °C	reduction in *L. monocytogenes* by 1.5 log_10_ CFU/g on the surface and 0.9 log_10_ CFU/g inside cheese at 4 °C; 3.7 log_10_ CFU/g on the surface at 10 °C	[155]
Chitosan film	0.5% and 1%	Shredded cabbage at 4 °C	Complete reduction in *L. monocytogenes* growth after 5 days in the presence of 0.5% chitosan film and 4 days in the presence of 1% chitosan film	[156]
Chitosan film with essential oils	1% (with 0.2% essential oils)	Shredded cabbage at 4 °C	Enhanced antimicrobial activity against *L. monocytogenes* compared to chitosan alone; complete inhibition after 6 days.	
Chitosan film	1%	Shredded black radish at 4 °C	Immediate reduction of 2.6–3.1 log_10_ CFU/g of *L. monocytogenes* after chitosan addition	[157]
Chitosan film with thyme oil	1% with 0.2% thyme oil	Shredded black radish at 4 °C	Reduction in *L. monocytogenes* by 2.1–2.4 log_10_ CFU/g	
Chitosan with 460 nm LED illumination	1.0% chitosan + 460 nm LED illumination at 2.4 kJ/cm^2^	Fresh-cut melon4 °C and 10 °C	Reduction in *L. monocytogenes* by 3.5 log_10_ CFU/cm^2^ at 4 °C and 10 °C	[154]
Chitosan film	1%	Extra thick bologna slices at 10 °C	Reduction in *L. monocytogenes* by 2 log_10_ CFU/bologna disc	[146]
Chitosan film with oregano	1% (with 1% and 2% oregano)	Extra thick bologna slices at 10 °C	Reduction in *L. monocytogenes* by 3.6 to 4 log_10_ CFU/bologna disc	
Chitosan film	1%	Ground meat at 4 °C	3 log_10_ reduction in *L. monocytogenes* population on Day 12	[158]
Chitosan film Nano-emulsions with rosemary	1% (with 1.56% rosemary nano-emulsions)	Ground meat at 4 °C	1 log_10_ reduction in *L. monocytogenes* population with RNE on Day 7	
Chitosan in hummus	0.5%	Hummus at 4, 10, and 25 °C	Reduction in *L. monocytogenes* by 2.0 log_10_ CFU/g at 4 °C for 28 days, 1.1 log_10_ CFU/g at 10 °C for 21 days, and 0.7 log_10_ CFU/g at 25 °C for 7 days	[159]
Chitosan in hummus	1%	Hummus at 4, 10, and 25 °C	Reduction in *L. monocytogenes* by 2.3 log_10_ CFU/g at 4 °C for 28 days, 2.0 log_10_ CFU/g at 10 °C for 21 days, and 1.1 log_10_ CFU/g at 25 °C for 7 days	
Chitosan in hummus with garlic	0.5% (with 1% garlic)	Hummus at 4, 10, and 25 °C	Reduction in *L. monocytogenes* by log_10_ 2.1 CFU/g at 4 °C for 28 days, 1.6 log_10_ CFU/g at 10 °C for 21 days, and 0.7 log_10_ CFU/g at 25 °C for 7 days	
Chitosan in hummus with garlic	1% (with 1% garlic)	hummus at 4, 10, and 25 °C	Reduction in *L. monocytogenes* by 2.7 log_10_ CFU/g at 4 °C for 28 days, 2.1 log_10_ CFU/g at 10 °C for 21 days, and 1.6 log_10_ CFU/g at 25 °C for 7 days	

**Table 2:** Antimicrobial effect of different chitosan applications against *Staphylococcus aureus* in laboratory media and food.

Chitosan Form	Antimicrobial Concentration	Medium/Food Matrix	Observed Antimicrobial Effect	Reference
Chitosan coated corona-treated polypropylene film	2%	Test strain inoculated nutrient broth on the surface of film	Reduction in *S. aureus* by 3.8 log during 24 h	[182]
Chitosan-propolis coated corona-treated polypropylene film	2% (with 200 mg/mL ethanolic extract of propolis)	Test strain inoculated nutrient broth on the surface of film	Reduction in *S. aureus* by 4.8 log during 24 h	
Different molecular weight (MW) chitosan	1%	BHI medium	Low-MW chitosan was more effective with greater inhibition zones against *S. aureus* compared to high MW chitosan	[122]
Different molecular weight chitosan	0.1%	Muller–Hinton broth	Reduction in *S. aureus* by 6.02–4.97 log_10_ CFU/g with high molecular weight; 5.08–4.21 log_10_ CFU/g with low molecular weight	[28]
Chitosan	0.5%	Muller–Hinton broth	Reduction in *S. aureus* by 6.02–4.97 log_10_ CFU/g after 24 h	[183]
Chitosan films with nisin	1% (with nisin at 51,000 IU/g)	Mueller–Hinton agar	Markedly high antimicrobial activity (inhibition zones) against *S. aureus*	[174]
Chitosan films with garlic oil	1% (with oil at 100 µL/g)	Mueller–Hinton agar	Even higher antimicrobial activity against *S. aureus* (inhibition zones) compared to chitosan films with nisin	
Chitosan	0.5% and 1%	White cheese solution at 4 °C	Reduction in *S. aureus* by 6 log_10_ CFU/g after 5 days at 0.5%; reduction of *S. aureus* by 6 log_10_ CFU/g after 1 days at 1%;	[171]
Chitosan	0.5, 1.0, and 2.0%	oyster	Reduction in *S. aureus* by 3.8, 2.1, 3.85 log_10_ CFU/mL compared to untreated control after 12-day	[184]
Chitosan	2%	Chicken balls at 3 °C	Reduction in *S. aureus* by 3.1 log_10_ CFU/g 12 days during storage.	[185]
Chitosan	2%	Fresh beef meat at ambient temperature	Reduction in *S. aureus* by 2.7 log_10_ CFU/g	[173]
Chitosan	2%	Frozen beef meat at ambient temperature and 4 °C	Reduction in *S. aureus* by 3.6 log_10_ CFU/g at 4 °C and 2.8 log_10_ CFU/g at ambient temperature	

**Table 3:** Antimicrobial effect of different chitosan applications against *E. coli* O157:H7 in foods.

Chitosan Form	Antimicrobial Concentration	Medium/Food Matrix	Observed Antimicrobial Effect	Reference
Chitosan	0.05–0.1%	Apple juice	Survival of *E. coli* O157:H7 was extended at 4 °C	[74]
Chitosan with 460 nm LED illumination	1.0% chitosan + 460 nm LED 1.3 kJ/cm^2^)	Fresh-cut melon at 4 °C and 10 °C	Reduction in *E. coli* O157:H7 by 3.5 log_10_ CFU/cm^2^ at 4 °C and 3.3 log_10_ CFU/cm^2^ at 10 °C	[153]
Chitosan with or without ZnO nanoparticles	2.5% (ZnO nanoparticles ≥ 0.0125%)	White brined cheese	Reduction in *E. coli* O157:H7 by 2.5 and 2.8 log_10_ CFU/g at 4 °C; 1.9 and 2.1 log_10_ CFU/g at 10 °C	[198]
Chitosan film with oregano	1% (with 1% and 2% oregano)	Extra thick bologna slices at 10 °C	Reduction in *E. coli* O157:H7 by 3 log_10_ CFU	[146]
Chitosan	2%	Iranian traditional ready-to-barbecue chicken meat cubes at 3 °C	Reduction in *E. coli* O157:H7 by 0.57 log_10_ CFU/g at 3 °C	[199]
Chitosan with oregano	2% (with 15% oregano oil)	Iranian traditional ready-to-barbecue chicken meat cubes at 3 °C	Reduction in *E. coli* O157:H7 by 1.21 log_10_ CFU/g at 3 °C	
Chitosan in hummus	0.5%	Hummus at 4, 10, and 25 °C	Reduction in *E. coli* O157:H7 by 2.7 log_10_ CFU/g at 4 °C for 28 days, 1.7 log_10_ CFU/g at 10 °C for 21 days, and 2.4 log_10_ CFU/g at 25 °C for 7 days	[159]
Chitosan in hummus	1%	Hummus at 4, 10, and 25 °C	Reduction in *E. coli* O157:H7 by 3.5 log_10_ CFU/g at 4 °C for 28 days, 2.3 log_10_ CFU/g at 10 °C for 21 days, and 2.2 log_10_ CFU/g at 25 °C for 7 days	
Chitosan in hummus with garlic	0.5% (with 1% garlic)	Hummus at 4, 10, and 25 °C	Reduction in *E. coli* O157:H7 by 3.2 log_10_ CFU/g at 4 °C for 28 days, 1.9 log_10_ CFU/g at 10 °C for 21 days, and 2.5 log_10_ CFU/g at 25 °C for 7 days	
Chitosan in hummus with garlic	1% (with 1% garlic)	Hummus at 4, 10, and 25 °C	Reduction in *E. coli* O157:H7 by 3.1 log_10_ CFU/g at 4 °C for 28 days, 2.6 log_10_ CFU/g at 10 °C for 21 days, and 3.1 log_10_ CFU/g at 25 °C for 7 days	
Commercial edible chitosan coating	2.5%	Mini-Roma cultivar tomatoes	Reduction in *E. coli* O157:H7 by 2.4 log_10_ CFU/g	[196]
Commercial edible chitosan coating with a lytic bacteriophage	2.5%	Mini-Roma cultivar tomatoes	Reduction in *E. coli* O157:H7 by 4.2 log_10_ CFU/g	
Chitosan-based antimicrobial solutions	5% (with 2% each of acetic, lactic and levulinic acids and 4% lauric arginate acid)	Marinades on beef top round steaks at 4 °C	Reduction in *E. coli* O157:H7 by 3.5 log_10_ CFU/cm^2^	[200]
Chitosan	0.4% (with 6.73 log_10_ *P. acidilactici*)	Meatballs	Reduction in *E. coli* O157:H7 by 1.7 log_10_ CFU/g during 10 days	[201]
Chitosan with *Pediococcus acidilactici*	0.4% (with 6.73 log_10_ *P. acidilactici*)	Meatballs at 4 °C	Reduction in *E. coli* O157:H7 by 2.2 log_10_ CFU/g during 10 days	
Chitosan (CH) with postbiotics (P) of *Pediococcus acidilactici*	0.5 and 1% (with 50–100% postbiotics)	Vacuum-packaged frankfurters at 4 °C	Reduction in *E. coli* O157:H7 in 0.5% CH + 50% P, 0.5% CH + 100% P, 1% CH + 50% P, and 1% CH + 100% P samples by 1.58, 1.62, 1.70, and 1.69 log_10_ CFU/g	[202]

**Table 4:** Antimicrobial effect of different chitosan applications against *Salmonella* spp. in foods.

Chitosan Form	Antimicrobial Concentration	Medium/Food Matrix	Observed Antimicrobial Effect	Reference
Chitosan	1%	Guacamole	Reduction in Salmonella by 0.5 log_10_ CFU/g for 7 days	[205]
Chitosan	1%	Fresh lean beef at 4 °C	Reduction in Salmonella by 1 log_10_ CFU/cm^2^ for 32 days	[207]
Chitosan with bacteriocin produced by *Carnobacterium maltaromaticum*	1% (with 1280 AU/mL purified bacteriocin)	Fresh lean beef at 4 °C	Reduction in Salmonella by 2 log_10_ CFU/cm^2^ for 32 days	
Chitosan coating with allyl isothiocyanate (AIT)	2% (with AIT at 60 μL/mL)	Fresh cantaloupes	Reduction in Salmonella by >5 log_10_ CFU/cm^2^ for 14 days	[208]
Chitosan film with 1,8-cineole (CIN)	1.5% (with 2, 3, 4% 1,8-cineole)	Model food surface (agar gel)	Reduction in Salmonella by 3 log_10_ CFU/cm^2^ for 7 days at 2 or 3% CIN and no growth of *Salmonella* from day 0 till day 7	[209]
Chitosan with 460 nm LED illumination	1.0% chitosan + 460 nm LED 1.3 kJ/cm^2^	Fresh-cut melon	Reduction in Salmonella by 0.9–1.1 log_10_ CFU/cm^2^	[153]
Chitosan with 460 nm LED illumination	1.0% chitosan + 460 nm LED illumination at 0.6–0.8 kJ/cm^2^	Fresh-cut melon	Reduction in Salmonella by 2.5 log_10_ CFU/cm^2^	[154]
Chitosan in hummus	0.5%	Hummus at 4, 10, and 25 °C	Reduction in Salmonella by 2.6 log_10_ CFU/g at 4 °C for 28 days, 1.5 log_10_ CFU/g at 10 °C for 21 days, and 2.1 log_10_ CFU/g at 25 °C for 7 days	[159]
Chitosan in hummus	1%	Hummus at 4, 10, and 25 °C	Reduction in Salmonella by 2.9 log_10_ CFU/g at 4 °C for 28 days, 2.1 log_10_ CFU/g at 10 °C for 21 days, and 2.2 log_10_ CFU/g at 25 °C for 7 days	
Chitosan in hummus with garlic	0.5% (with 1% garlic)	Hummus at 4, 10, and 25°C	Reduction in Salmonella by 2.7 log_10_ CFU/g at 4 °C for 28 days, 1.4 log_10_ CFU/g at 10 °C for 21 days, and 1.1 log_10_ CFU/g at 25 °C for 7 days	
Chitosan in hummus with garlic	1% (with 1% garlic)	Hummus at 4, 10, and 25°C	Reduction in Salmonella by 2.8 log_10_ CFU/g at 4 °C for 28 days, 2.5 log_10_ CFU/g at 10 °C for 21 days, and 1.3 log_10_ CFU/g at 25 °C for 7 days	

## Data Availability

No new data were created or analysed in this study.
